# Value-Based Integrated Care: A Systematic Literature Review

**DOI:** 10.34172/ijhpm.2024.8038

**Published:** 2024-02-19

**Authors:** Evelien S. van Hoorn, Lizhen Ye, Nikki van Leeuwen, Hein Raat, Hester F. Lingsma

**Affiliations:** Department of Public Health, Erasmus MC, Erasmus University Medical Centre, Rotterdam, The Netherlands.

**Keywords:** Value-Based Healthcare, Delivery of Healthcare, Systematic Review, Integrated care, Facilitators & Barriers, Effects Evaluation

## Abstract

**Background:** Healthcare services worldwide are transforming themselves into value-based organizations. Integrated care is an important aspect of value-based healthcare (VBHC), but practical evidence-based recommendations for the successful implementation of integrated care within a VBHC context are lacking. This systematic review aims to identify how value-based integrated care (VBIC) is defined in literature, and to summarize the literature regarding the effects of VBIC, and the facilitators and barriers for its implementation.

**Methods:** Embase, Medline ALL, Web of Science Core Collection, and Cochrane Central Register of Controlled Trails databases were searched from inception until January 2022. Empirical studies that implemented and evaluated an integrated care intervention within a VBHC context were included. Non-empirical studies were included if they described either a definition of VBIC or facilitators and barriers for its implementation. Theoretical articles and articles without an available full text were excluded. All included articles were analysed qualitatively. The Rainbow Model of Integrated Care (RMIC) was used to analyse the VBIC interventions. The quality of the articles was assessed using the Mixed Methods Appraisal Tool (MMAT).

**Results:** After screening 1328 titles/abstract and 485 full-text articles, 24 articles were included. No articles were excluded based on quality. One article provided a definition of VBIC. Eleven studies reported—mostly positive— effects of VBIC, on clinical outcomes, patient-reported outcomes, and healthcare utilization. Nineteen studies reported facilitators and barriers for the implementation of VBIC; factors related to reimbursement and information technology (IT) infrastructure were reported most frequently.

**Conclusion:** The concept of VBIC is not well defined. The effect of VBIC seems promising, but the exact interpretation of effect evaluations is challenged by the precedence of multicomponent interventions, multiple testing and generalizability issues. For successful implementation of VBIC, it is imperative that healthcare organizations consider investing in adequate IT infrastructure and new reimbursement models.

**Systematic Review Registration:** PROSPERO (CRD42021259025).

## Background

 Nowadays, integrated care is often seen as the future direction for the development of healthcare systems driven by the aging population, increase in patients with comorbidity and the associated increase in healthcare expenditure.^1,2^ Integrated care can take many different forms, and there is no unifying definition.^1,3-6^ The definition of integrated care is dependent on the different views and expectations of the various stakeholders.^7,8^ What unifies the different definitions, however, is that integrated care is an approach to overcome care fragmentation leading to improved efficacy of care, patient outcomes and experiences with care.^8,9^

 To successfully implement integrated care, it is essential to understand its complexity. Different taxonomies have been developed to guide healthcare professionals, managers, policy-makers, researchers and other stakeholders to differentiate and analyse the different forms of integrated care.^7,8,10^ Those taxonomies typically describe the type of integration (ie, professional and organizational), the level at which integration occurs (ie, macro-, meso-, and micro-), the degree of integration (ie, from informal linkages to more managed care coordination and fully integrated teams or organizations), the process of integration (ie, how integrated care is organized and managed) and the breadth of integration (ie, to a whole population or specific group).^8^

 In order to provide more sustainable healthcare, healthcare services worldwide are transforming themselves into value-based organizations.^11,12^ By implementing value-based healthcare (VBHC), healthcare organizations aim to maximize value for patients by achieving the best patient outcomes at the lowest possible costs.^13,14^ Integrated care is an important aspect within the VBHC framework; integrated practice units (IPUs) and the integration of care delivery across multiple separate facilities are two of the core pillars of VBHC.^13^ The implementation of IPUs and the integration of care delivery across multiple facilities should reduce duplication of efforts, delays and inefficiencies in the healthcare process.^13^ In an IPU, care is delivered by a dedicated, multidisciplinary team who takes responsibility for the full cycle of care for a specific condition, encompassing outpatient, inpatient and rehabilitative care, as well as supporting services.^13^ Members of an IPU see themselves as one organizational unit and share a common administrative and scheduling structure. An essential element of integrated care within the VBHC framework, described in theory, is that IPUs routinely measure outcomes, cost, care processes, and patient experience using a common platform and accept joint accountability for the results.^13,15^

 In the current literature, several reviews have been performed to provide healthcare organizations with practical and evidence-based recommendations for the successful implementation of integrated care. Reviews have summarized the literature on how integrated care is implemented,^1^ the facilitators and barriers for its implementation,^16,17^ and its effectiveness.^2,18,19^ Until now, no overview of the literature exists to identify those elements for integrated care within a VBHC context.

 This systematic review aims to provide practical evidence-based recommendations for the successful implementation of integrated care within a VBHC context. To achieve this, we aim to identify how integrated care within a VBHC context, in other words value-based integrated care (VBIC), is defined in the current literature. Furthermore, we aim to summarize the results of evaluations of the effects of VBIC, and to summarize the literature regarding the facilitators and barriers of its implementation.

## Methods

###  Search Strategy 

 This review was conducted in line with the Preferred Reporting Items for Systematic Reviews and Meta-Analysis (PRISMA) guidelines.^20^ The electronic databases Embase, Medline ALL, Web of Science Core Collection, and Cochrane Central Register of Controlled Trials were systematically searched for relevant articles from the date of inception of each database until January 15, 2022. To identify publications that reported on VBIC, the literature search included search terms related to both VBHC and integrated care. Since there is no unambiguous definition for integrated care, synonyms such as comprehensive care, coordinated care and multidisciplinary care were included within the search. Synonyms and other terms related to VBHC were also incorporated within the search. The search terms were adequately adjusted for each database and included both registered and non-registered index terms. Further details of the search strategy are available in the Supplementary Material. The protocol was registered in the PROSPERO database (registration number: CRD42021259025).

###  Eligibility Criteria and Article Selection

 To be eligible for this review, publications had to meet the following criteria: (1) description of an empirical study, (2) covering a healthcare context, (3) written in English or Dutch, (4) description of VBHC and integrated care (including any spelling variation and synonyms) in the introduction or method section, (5) provide a definition for VBIC, describe the effects of VBIC or mention facilitators and barriers for its implementation. Theoretical articles (eg, commentaries) and articles without an available full text (eg, conference abstracts) were excluded. One exception was made to the eligibility criteria. Non-empirical studies were included if they provided a definition of VBIC or mentioned facilitators and barriers for its implementation to ensure all relevant publications were included within this review.

 All articles were screened against the eligibility criteria in two phases; first, the titles and abstracts were screened, followed by the full-text screening. Both the title and abstract, and full-text screening was performed by two independent reviewers. When there were conflicts about whether an article met the inclusion criteria, a third reviewer was consulted for a third opinion and discrepancies were discussed until consensus was reached. Deduplication was conducted in Endnote and article screening was performed using *Covidence.*^21^

###  Quality Assessment

 The methodological quality of the included articles was appraised independently by two reviewers using the Mixed Methods Appraisal Tool (MMAT). The MMAT permits the appraisal of five different study designs; (1) qualitative research, (2) randomized controlled trials, (3) non-randomized studies, (4) quantitative descriptive studies, and (5) mixed methods studies.^22^ This allowed the use of one appraisal tool for all included studies within this systematic review. The MMAT consist of two screening questions and five questions per study design. All questions can be answered with “Yes,” “No,” or “Cannot tell.” Responding “No” or “Cannot tell” on the screening questions indicates that the study is not an empirical study and further appraisal may not be feasible or appropriate.^22^ This review, therefore, only assessed the methodological quality of the empirical studies. Articles of which three of the five questions related to the study design were answered with yes were characterized as having a good methodological quality. Any discrepancies in the quality assessment were resolved by consulting the third reviewer.

###  Data Extraction and Analysis

 All included studies were analysed qualitatively using a narrative approach by two independent reviewers and data were extracted on the following items: definition of VBIC, the study and intervention characteristics (eg, study design, population, and country), all outcome measures and results of the VBIC intervention, and the facilitators and barriers for its implementation. The VBIC interventions were categorized using the Rainbow Model of Integrated Care (RMIC).^23^ The RMIC distinguishes six integration dimensions (clinical, professional, organizational, system, functional, and normative integration). The first four dimensions describe the type of integration and level at which integrated care can occur: macro- (system) level, meso- (organizational and professional) level, and micro- (clinical) level. The other dimensions, functional and normative integration, describe the mechanisms, or in other words facilitators, that support the implementation of integrated care (Table 1).^23,24^

**Table 1:** Integrated Care Dimensions of the Rainbow Model of Integrated Care

**Level**	**Dimension**	**Description**
Micro	Clinical integration	The coordination of person-focused care in a single process across time, place and discipline.
Meso	Professional integration	Inter-professional partnerships based on shared competences, roles, responsibilities and accountability to deliver a comprehensive continuum of care to a defined population.
Meso	Organizational integration	Inter-organizational relationships (eg, contracting, strategic alliances, knowledge networks, and mergers), including common governance mechanisms, to deliver comprehensive services to a defined population.
Macro	System integration	A horizontal and vertical integrated system, based on a coherent set of (informal and formal) rules and policies between care providers and external stakeholder for the benefit of people and populations.
Micro, meso, macro	Functional integration	Key support functions and activities (ie, financial, management, and information systems) structured around the primary process of service delivery to coordinate and support accountability and decision-making between organizations and professionals in order to add overall value to the system.
Micro, meso, macro	Normative integration	The development and maintenance of a common frame of reference (ie, shared mission, vision, values, and culture) between individuals, professional groups and organizations.

## Results

###  Search Results 

 After deduplication, the combined search yielded 1328 unique articles. After the title and abstract screening phase, 485 records were screened on full text. After full text inspection 461 articles were excluded for the following reasons: the article was not in English or Dutch (n = 2), no full text was available (n = 27), the article did not mention VBHC and integrated care in the introduction or method section (n = 156), or the article was not empirical and/or did not describe a definition, facilitators and barriers or effects of VBIC (n = 276). At the end, 24 articles met the inclusion criteria (Figure).

**Figure:**
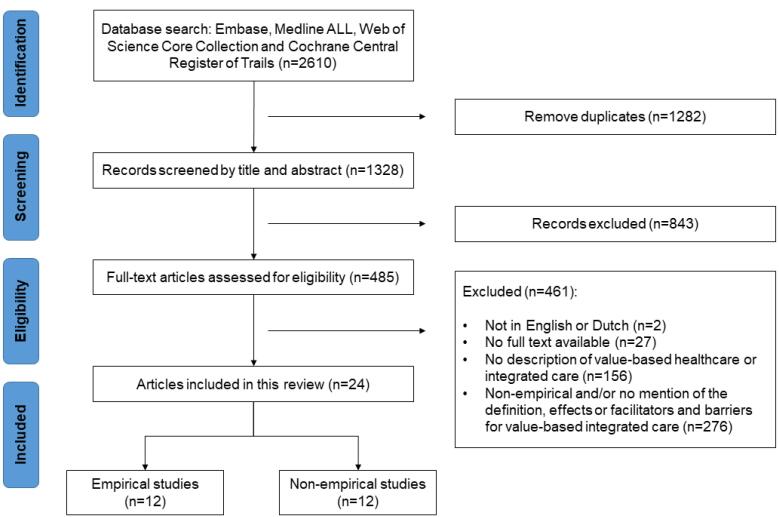
Flowchart Depicting the Article Selection.

###  Study Characteristics and Quality

 The included articles were published between 2013 and 2021. Seventy-one percent (n = 17) of all included studies were conducted in the United States, 8% (n = 2) in the Netherlands and the remaining 21% (n = 5) in Italy, Spain, Sweden, the United Kingdom, and Taiwan. Fifty percent (n = 12) of the included publications described an empirical study, of which almost all performed a quantitative analysis (n = 11). Quality assessment was performed for all empirical studies. Of these studies, seven were categorized as non-randomized studies,^25-31^ three as randomized controlled trails,^32-34^ one as qualitative study^35^ and one as a quantitative descriptive study.^36^ Almost all articles had a good methodological quality (Table 2). One article^36^ had a questionable methodological quality; almost all questions of the MMAT were answered with “No” or “Cannot tell.” This article was not excluded since it contained relevant information about the facilitators and barriers for VBIC.

**Table 2:** Methodological Quality of the Articles

**Authors, Country and Year**	**Screening Questions**		**Study Design Questions**				
	**1**	**2**	**1**	**2**	**3**	**4**	**5**
Dolce et al,^31^ USA, 2020	Yes	Yes	Yes	Yes	Yes	Cannot tell	Cannot tell
Lee et al,^34^ Taiwan, 2021	Yes	Yes	Yes	Yes	Yes	Yes	Yes
Price-Haywood et al,^33^ USA, 2021	Yes	Cannot tell	Yes	Yes	Cannot tell	Cannot tell	Yes
Fortmann et al,^30^ USA, 2020	Yes	Yes	Yes	Yes	Cannot tell	Cannot tell	Yes
Gabriel et al,^29^ United Kingdom, 2019	Yes	Yes	Yes	Yes	Yes	Cannot tell	Yes
Goretti et al,^26^ Italy, 2020	Yes	Yes	Yes	Yes	Cannot tell	Yes	Yes
Hernandez et al,^36^ USA, 2019	Cannot tell	Cannot tell	Yes	Cannot tell	No	No	Yes
Van Deen et al,^28^ USA, 2017	Yes	Yes	Yes	Yes	Cannot tell	Cannot tell	Yes
Van Veghel et al,^27^ The Netherlands, 2020	Yes	Yes	Yes	Yes	Yes	No	Yes
Wood et al,^32^ USA, 2016	Yes	Yes	Yes	Yes	Yes	Cannot tell	Yes

###  The Definition of Value-Based Integrated Care

 Within the included articles, one study provided an explicit definition of VBIC. Valentijn et al defined VBIC as “patients’ achieved outcomes and experience of care in combination with the amount of money spent by providing accessible, comprehensive and coordinated services to a target population.”^37^ Two other articles referred to this definition.^38,39^ All other articles did not specify or mention the term VBIC. Those articles used a combination of integrated care synonyms and VBHC to describe VBIC. The most commonly mentioned integrated care synonyms were IPU’s,^26,29,35,36,39-44^ multidisciplinary teams,^25,38,45,46^ multidisciplinary or interdisciplinary care,^28,31,33^ team-based care,^30,47^ and working together across disciplines or institutions.^27,48^

###  Interventions and Effects of Value-Based Integrated Care

####  Value-Based Integrated Care Interventions

 Twelve articles described the implementation and evaluation of an integrated care intervention within a VBHC context (Table 3). The VBIC interventions consisted of multiple components, targeted different patient populations and occurred in different settings. The interventions were implemented in primary care,^30,31,33,34^ primary and secondary care,^36^ secondary care,^27-29,32^ or tertiary care.^25,26,35^ According to the RMIC, the VBIC interventions can be classified as clinical,^30,31,33,34^ professional,^26,28,29,32,35,36^ organization,^27^ and system integration.^25^

**Table 3:** A Description of the Interventions And Quantitative Results of the Value-Based Integrated Care Interventions

**Authors, Country, and Year**	**Study Design**	**Patient Population**	**Program Description**		**Results**	
				**Intervention**	**(Patient) Outcomes**	**Cost**
Dolce et al,^31^ USA 2020	Method: non experimental pre-test/post-test (cohort study) Sample size: N = 31 Control group: no	Patients aged 65 years and older with hypertension and/or type 2 diabetes.	Implementation of the NPD model for primary care with monthly wellness visits by the nurse practitioner. Intervention elements: (1) revision of patient workflow processes, and (2) monthly wellness visits.	Level of integration: clinical Setting: primary care	**After implementation** *Clinical outcomes* ↓ systolic BP (*P *= .053); ↔ diastolic BP (*P *= .550); ↓ weight (*P *< .001); ↓ BMI (*P *= .011); ↓ HbA1c (*P *= .037) *Patient-reported outcomes* ↑ patients’ self-assessed confidence in meeting goals (*P *< .001)	**After implementation** *Process evaluation* ↓ proportion of patients who received advanced care planning (*P *= .002)
Lee et al,^34^ Taiwan 2021	Method: prospective randomized controlled trail Sample size: N = 398 Control group: yes	Patients older than 65 years with at least three chronic medical conditions.	Evaluation of an integrated multi-domain intervention. Intervention elements: (1) 16 a-h sessions per year comprising communal physical exercise, cognitive training, nutrition and disease education, and (2) individualized treatment by specialist in integrated geriatric care.	Level of integration: clinical Setting: primary care	**Intervention compared to usual care** *Clinical outcomes* ↔ no difference in serum biomarkers after 12 months *Patient-reported outcomes * ↑ physical component summary score (*P *= .010); ↑ physical functioning (*P *= .084); ↓ physical role limitation (*P *= .0016); ↓ bodily pain (*P *= .19);↑ general health (*P *= .028); ↑ mental component summary (*P *= .12); ↑ vitality (*P *= .0048); ↑ social functioning (*P *= .94); ↓ emotional role limitation (*P *= .091); ↑ mental health (*P *= .046); ↑ value-based metric scores on ICHOM Standard Set for Older Person (*P *= .0031); ↓ global cognitive impairment (*P *< .0001)	
Price-Haywood et al,^33^ USA 2021	Method: a modified stepped-wedge cluster randomized clinical trial design Sample size: not mentioned Control group: yes	Patients with co-morbid chronic non-cancer pain with depression or anxiety.	Implementation of BHI-CCM in addition to an EMR CDS for safe pain management. Intervention elements: (1) routine screening of patients for psychiatric conditions, (2) patient education and self-management support, (3) medication management, (4) clinical monitoring of response to treatment, (5) psychotherapy, (6) standardized follow up, (7) formal stepped care for systematic adjustment of care plans until treatment goals are achieved, and (8) physician supervision.	Level of integration: clinical Setting: primary care	**No results mentioned** *Patient-reported outcomes* PHQ-9 depression; GAD-7 anxiety; PROMIS-10 global health; Social determinants of health; PEG-3; COMM-9 *Healthcare provider experience* Provider experience with mental healthcare management	**No results mentioned** *Healthcare utilization* inpatient hospitalizations ED use *Process indictors* % morphine prescribed high dose (>50 mg); % morphine prescribed very high dose (>90 mg); % specialty referrals; % pain agreements; % urine drug screening; % naloxone documented; % non-opioid prescriptions
Fortmann et al,^30^ USA 2020	Method: pragmatic, quasi-experimental case control Sample size: N = 475 Control group: yes	Patients with diabetes and cardiovascular risk factors.	Implementation of a CMC-TI. Intervention elements: (1) a registered nurse/certified diabetes educator care manager, (2) medical assistant health coach, (3) registered nurse depression care manager, (4) utilized electronic medical record-based risk stratification reports, (5) standardized decision-support tools, (6) live and remote tailored treatments, and (7) coaching to manage care.	Level of integration: professional Setting: primary care	**CMC-TI group compared to usual care** *Clinical outcomes* ↓ HbA1c (*P *= .011); ↔ no difference LDL-C or systolic BP *Patient-reported outcomes* ↑ healthful eating (*P* < .05); ↑ exercise (*P* < .05); ↑ blood glucose monitoring (*P* < .05); ↑ foot-checking (*P* < .05); ↔ self-reported medication adherence; ↓ diabetes distress over 1 year (*P* < .05)	**CMC-TI group compared to usual care** *Healthcare utilization* ↓ percentages of patients with ≥1 inpatient encounter (*P* = .001); ↓ mean number of ED visits (*P* = .013); ↓ mean number of inpatient hospitalizations (*P* = .004); ↓ mean inpatient encounters (*P* < .001) *Cost* ↓ total healthcare cost (inpatient + ambulatory) (*P* = .002); *Process evaluation; *↑ follow-up/care coordination (*P* < .05); ↑ support for patient activation (*P* < .05); ↑ self-management (*P* < .05); ↑ delivery system design/decision support (*P* < .05)
Gabriel et al, ^29^ United Kingdom 2019	Method: retrospective cross-sectional cohort study Sample size: N = 50 Control group: yes	Patients with primary hip osteoarthritis who underwent a routine primary total hip replacement.	Evaluation of two different care pathways; one a traditional model with multiple entry points and without standardization, and one with an intentionally designed standardized multidisciplinary pathway (IPU). Intervention elements (IPU): (1) specialist integrated MSK unit, (2) a linear pathway, and (3) triage by the extended scope triaging physiotherapist.	Level of integration: professional Setting: secondary care	**IPU compared to usual care ** *Clinical outcomes* ↔ 100% survival in both cohorts *Patient-reported outcomes* ↔ no difference in PROMs across both cohorts	**IPU compared to usual care ** *Cost* ↓ the IPU generated lower costs (*P* > .05)
Goretti et al,^26^ Italy 2020	Method: observational cohort study Sample size: N = 2122 Control group: no	Patients with obesity.	Implementation of VBHC strategy associated with the ERAS protocol for patients with obesity. Intervention elements: (1) IPU implementation, (2) value stream mapping, and (3) redesign of both clinical and organizational processes.	Level of integration: professional Setting: secondary care	**After implementation(no ** ***P*** ** values mentioned; no comparison with before implementation)** *Clinical outcomes* - 74% mean EWL after 1 year - 82% mean EWL after 3 years - 81% of patients with type 2 diabetes obtaining normal HbA1c values - 76% of patients recovered from sleep apnoea - 56% of patients recovered from hypertension - 0% mortality within 30 days of surgery - 1.8% morbidity within 30 days of surgery - 0.4% readmission and reoperation rate within 30 days of surgery - 77.5% of patients experienced no pain at all during hospitalization - 22.5% of patients reported pain or discomfort during hospitalization - 28% of patients reported mild nausea during hospitalization - 11% of patients reported vomiting during hospitalization - 61% of patients reported no symptoms during hospitalization *Patient-reported outcomes* - 77% of patients reported to work better and more than before the procedure - 89% were able to practice physical activities - 52% reported a longer training time - 92% buy clothes everywhere and not in special shops for oversized costumers - 90% graded their sexual life as good - 48% reported an improvement in sexual life	**After implementation(no ** ***P*** ** values mentioned; no comparison with before implementation)** *Process evaluation* - patients spent 40% less time at the hospital completing all the exams in a single morning - 92% of patients had oral fluid uptake 2-8 h before surgery - 100% of patients adoption preoperative fluid management and PONV prophylaxis - standardized anaesthetic protocol was fully applied - 0.2% postoperative intensive care admittance - average length of stay of 2.1 days - 82% response to follow-up phone calls from case manager - 83% compliance to 1-year follow up visit with surgeon *Cost* ↔ additional costs associated with the intervention were compensated by the additional revenue obtained
Hernandez et al,^36^ USA 2019	Method: cohort study Sample size: not mentioned Control group: no	Active Navy and Marine Corps personnel, their dependents as well as retirees with low back pain, osteoarthritis, diabetes or pregnancy.	Implementation of 4 IPU’s (diabetes, low back pain, osteoarthritis and pregnancy) in Navy medicine.	Level of integration: professional Setting: primary and secondary care	**After implementation (no ** ***P *** **values mentioned)** *Clinical outcomes* ↓ mean morphine use (low back pain); ↓ 2.5% lower average HbA1c (diabetes) *Patient-reported outcomes* ↓ disability (low back pain); ↑ quality of life (diabetes); ↑ ease of disease management (diabetes); ↑ average hip disability and knee injury osteoarthritis outcome score (osteoarthritis)	**After implementation (no ** ***P*** ** values mentioned)** *Healthcare utilization* ↓ 60% less time in physical therapy (low back pain); ↑ greater use of behavioural health and nutrition resources (high-risk pregnancy) *Process indicators* ↓ time to diagnosis (low back pain); ↑ % of patients enrolled with appropriate imaging (osteoarthritis) *Cost* ↓ quarterly cost (over all IPU’s)
Van Deen et al,^28^ USA 2017	Method: case control Sample size: N = 237 Control group: yes	Patients with IBD.	Implementation of VBHC program for patients with IBD. Intervention elements: (1) constant monitoring of health outcomes, (2) highly coordinated care pathways, (3) patient education, and (4) task differentiation between providers.	Level of integration: professional Setting: secondary care		**VBHC group compared to usual care** *Healthcare utilization* ↓ office visits (*P* = .41); ↓ office visits with a gastroenterologist (*P* = .32); ↓ ED visits (*P* = .44); ↓ hospitalizations (*P* = .71); ↓ colonoscopies (*P* = .45); ↓ upper endoscopies (*P* = .012); ↓ surgeries (*P* = .49); ↑ complete blood count tests(*P* = .23); ↑ liver enzyme tests (*P* = .23); ↑ C-reactive protein test (*P* = .33); ↑ ESR test (*P* = .16); ↓ stool calprotectin test (*P* = .015); ↑ clostridium difficile stool test (*P* = .77); ↓ radiography (*P* = .61); ↓ CT scans (*P* = .090); ↓ MR scans (*P* = .25); ↓ ultrasounds (*P* = .83); ↔ medication use; ↓ relapses (*P* = .70) *Cost* *↓ *average annual costs (*P* = .24)
Van Veghel et al,^27^ The Netherlands 2020	Method: observational cohort study Sample size: N = 1475 Control group: no	Patients with CAD treated with a CABG or PCI.	Evaluation of a pilot study to enhance regional integration between two hospitals (SJG Weert and Catharina cardia centre). Intervention elements: (1) improved information and communication within and between hospitals, (2) new protocol for patients’ discharge, (3) modified patient brochures, (4) daily discussion sessions and frequent multidisciplinary meetings, (5) increase in consultant capacity, (6) planning modification at outpatient clinic, (7) introduction of outpatient clinic prior to complicated procedures and for specific patient groups, (8) introduction of time-outs in catheterization lab, and (9) change of discharge policy.	Level of integration: organization Setting: secondary care	**After implementation** *Clinical outcomes (both PCI or CABG) * ↓ mortality; ↓ complications; ↓ event-free survival (short term); ↑ event-free survival in SJG Weert compared to all other referring hospitals in 2014-2016 (*P* = .046); ↔ event-free survival between SJG Weert and other referring hospitals in 2011–2013 (*P* = .653) *Patient experiences* ↑ patient information and education (*P* = .013); ↔ expectation management (*P* = .127); ↔ alignment between both hospital (*P* = .214); ↔ communication with GP (SJG Weert *P* = .086, Catharina *P* = .189); ↔ duration to approach and pathway (SJG Weert *P* = .729, Catharina *P* = .134); ↑ quality of care at SJG Weert (*P* = .007); ↔ quality of care at Catharina (*P* = .057); ↑ admission and stay at SJG Weert (*P* = .32); ↔ admission and stay at Catharina (*P* = .155); ↑ general grade SJG Weert (*P* = .007); ↔ general grade Catharina (*P* = .070); ↑ personal contact between patient and physician at SGJ Weert (*P* = .024); ↑ personal contact between patient and physician at Catharina (*P* = .031)	
Wood et al,^32^ USA 2016	Method: retrospective cross-sectional cohort study Sample size: N = 200 Control group: yes	Patients with ischemic stroke and TIA.	Implementation of an evidence-based intervention consisting of a collaborative APNs and hospitalist physician model of care for patients on the hospital’s stroke unit. Intervention elements: (1) unit-based assignment of one full-time APN to be available on the stroke unit, (2) collaborative medical decision making shared by the APNs and multiple collaborating hospitalist physicians, and (3) participation by the APNs in the stroke unit multidisciplinary team meetings.	Level of integration: organization Setting: secondary care	**Collaborative APN care model compared to usual care** *Patient experience* ↑ overall quality of hospital stay (*P* = .014); ↑ overall teamwork among staff (*P* = .046); ↔ no difference in overall quality of care (*P* = 1.000)	**Collaborative APN care model compared to usual care** *Hospital utilization* ↔ no difference in mean length of stay (stroke *P* = .953, TIA *P* = .316) ↔ no difference in unplanned all-cause 30 day readmissions (*P* = .630) *Process indicators (quality measures)* ↔ no difference in achievement on the measure antiplatelet added by day 2 (*P* = .059); ↔ no difference in achievement on the measure DVT prophylaxis (*P* = .0537); ↔ no difference in achievement on the measure rehabilitation assessment (*P* = 1.000); ↑ higher achievement on the measure statin at discharge (*P* = .015); ↔ no difference in in achievement on the measure anticoagulation for atrial fibrillation (*P* = .444)
Regueiro et al,^25^ USA 2018	Method: quasi-experimental, time-interrupted study Sample size: N=322 Control group: no	Patient with CD or UC.	Implementation of an IBD specialty medical home. Intervention elements: (1) team-based care with physician extenders, nurse coordinators, schedulers, social workers, and dietitians, (2) effective care coordination, (3) tracking of process and outcome metrics of interest, (4) appropriate use of technology to enhance clinical care, and (5) care access, after-hours care, and follow-up care after emergency room visits and hospitalizations.	Level of integration: system Setting: secondary care	**After implementation** *Patient-reported outcomes* ↓ disease activity (CD *P* = .002, UC *P* = .0003); ↓ depression (*P* < .0001); ↓ anxiety (*P* = .02); ↑ quality of life (*P* < .0001)	**After implementation** *Healthcare utilization * ↓ number of ED visits (*P* < .0001); ↓ number of hospitalizations (*P* < .008); ↑ number of intestinal resections (*P* = .22); ↓ number of radiographic studies (*P* = .06); ↓ number of endoscopic procedures (*P* = .08)

Abbreviations: APN, advance practice nurse; BHI-CCM, integrated behavioral health collaborative care management; BMI, body mass index; BP, blood pressure; CABG, coronary artery bypass graft; CAD, coronary artery disease; CD, Crohn’s disease; CMC-TI, cardiometabolic care team intervention; COMM, Current Opioid Misuse Measure; CT, computed tomography; DVT, deep vein thrombosis; ED, emergency department; EMR CDS, electronic medical record clinical decision support; ERAS, Enhanced Recovery After Surgery; EWL, excess weight loss; GAD, General Anxiety Disorder; GP, general practitioner; HbA1C, glycosylated hemoglobin; IBD, inflammatory bowel disease; ICHOM, International Consortium for Health Outcomes Measurement; IPU, Integrated Practice Unit; LDL-C, low-density lipoprotein cholesterol; MR, magnetic resonance; MSK, musculoskeletal; PCI, percutaneous coronary intervention; PEG, Pain, Enjoyment, General Activity; PHQ, Patient Health Questionnaire; PONV, Post-Operative Nausea and Vomiting; PROMIS, Patient-Reported Outcomes Measurement Information System; SJG, St. Jans Gasthuis; TIA, transient ischemic attack; UC, ulcerative colitis; VBHC, value-based healthcare; NPD, Nurse Practitioner-Dentist; ESR, erythrocyte sedimentation rate.

####  Effects of Value-Based Integrated Care – Quantitative Analysis

 In 11 studies, a wide range of outcome measures was used to evaluate the effect of the VBIC intervention (Table 3). All articles analysed the effect of the VBIC intervention on multiple outcome measures. The outcome measures consisted of: (1) patient-reported outcomes (eg, quality of life, disease activity), (2) clinical outcomes (eg, HbA1c, weight, and mortality), (3) healthcare utilization (eg, emergency department [ED] visits, hospitalizations, and patient encounters), (4) cost of care, (5) patient experiences (eg, quality of care, satisfaction with care), and (6) process indicators (eg, proportion of patients that received care according to protocol). Almost all articles described a positive effect of the VBIC intervention on at least one of these outcome measures.

####  Effects of Value-Based Integrated Care – Qualitative Analysis

 One article^35^ reports the results of a qualitative evaluation. This study by Nilsson et al aimed to explore how participants experienced the implementation of VBHC at a Swedish University Hospital.^35^ A part of the intervention focused on increasing cooperation with other departments or care institutions within the care chain. This review focused on this part of the intervention, not the intervention as a whole. The participants noted that the increased cooperation across departments made it easier to obtain outcome measurements and to perform patient follow-ups. In addition, increased cooperation increased the participants understanding of different conditions treated at each department and of conditions for different patient populations. Furthermore, the intervention increased the awareness of cooperation between inpatient and outpatient care. The increase in cooperation contributed to increased accessibility for the patients to receive care at the right care level.^35^

###  Facilitators and Barriers for the Implementation of Value-Based Integrated Care

 Almost all articles (n = 19, 79%) described either a facilitator or barrier for the implementation of VBIC. Facilitators and barriers were found for all types and levels of integration (ie, clinical, professional, organizational, and system level). The various facilitators and barriers for the implementation of VBIC were characterized as either a functional or normative integration mechanism and grouped into nine different categories: (1) information technology (IT), (2) financing, (3) organizational culture and leadership, (4) workforce, (5) communication and coordination, (6) commitment, (7) clinical care, (8) education, and (9) quality improvement. Facilitators were most often mentioned in the categories of IT, financing, and communication and coordination (Table 4). Specifically, the most frequently reported facilitators were supportive IT (n = 8), a new reimbursement or payment model (n = 7) and leadership (n = 4). Barriers were mentioned most often in the categories of IT, financing and workforce. Commonly reported barriers were limited or insufficient IT (n = 8), current reimbursement or payment model (n = 7), and the required cultural change (n = 4).

**Table 4:** Facilitators and Barriers for the Implementation of Value-Based Integrated Care Categorized According to the Dimensions of the Rainbow Model of Integrated Care

	**Clinical Integration**	**Professional Integration**	**Organization Integration**	**System Integration**	**Functional Integration**	**Normative Integration**
**Facilitators for Value-Based Integrated Care**						
*Information technology*						
Advances in IT		^41^			✓	
Information technology tools, like electronic health records, e-referral systems and information systems to support the communication between patients and/or providers	^37^	^28,37,42^	^37^		✓	
Information technology infrastructure that allows clinical data connectivity, integration and care coordination		^47^	^48^		✓	
Integrated IT platform to measure outcomes and cost across patient pathways		^40^	^40,44^	^40^	✓	
*Financing*						
New reimbursement systems/payment models		^29,39-41,44,46^	^40,46^	^40,46^	✓	
Developing team-based payment models		^47^			✓	
Integrated delivery and finance system				^25^	✓	
*Organizational culture and leadership *						
Strong leadership, with skills in collaborative working, communication, motivation, and vision setting		^35^	^44^			✓
Well-defined leadership structure and process		^36,39^			✓	
Shared vision and alignment with mission		^36^	^48^			✓
Change in organizational culture, focus on continuous improvement			^44^			✓
*Workforce*						
An adequately staffed, well-trained and coordinated workforce		^47^			✓	
Ability to translate top-down strategy decisions to fit different local conditions		^35^				✓
*Communication and coordination *						
Create affiliations with appropriate providers and/or develop new partnerships		^41,46^	^46^	^46^		✓
Communication and coordination among stakeholders (healthcare providers, patients, board and management staff)	^31^	^26^				✓
Align incentives to support the delivery of coordinated care			^44^			✓
Align incentives between payers and providers				^25^		✓
Cooperation between different departments involved in the same patient journey		^35^				✓
Effective shared governance structure			^48^		✓	✓
*Commitment*						
Buy-in at clinical and managerial levels		^39^			✓	✓
Team commitment to the initiative’s central goals		^36^				✓
Mutual trust and respect among different providers	^37^	^37^	^37^			✓
Patients’ and caregivers’ involvement		^26^				✓
*Clinical care*						
High patient volumes *(clear IPU selection criteria)*		^36^			✓	
The existence of standardized, evidence-based clinical pathways		^36^			✓	
*Education*						
Setting up regular multidisciplinary conferences		^41^			✓	
Promoting education and awareness		^41,46^	^46^	^46^	✓	
Educating both providers and patients	^31^				✓	
*Quality improvement*						
Common set of clinical quality measures and protocols			^48^		✓	
Readily available outcome measures	^31^	^36^			✓	
Dedicate resources (human and financial) to make outcome measurement core business					✓	
Evaluate the impact on patient and providers satisfaction, quality improvement and cost savings		^46^	^46^	^46^	✓	
Resource investment in order to allow data collection		^39^			✓	
Choosing a duration of care cycles that enables useful feedback to clinicians		^36^			✓	
**Barriers for Value-Based Integrated Care**						
*Information technology*						
Limited current IT infrastructure		^36,40^	^40^	^40^	✓	
Limited access to electronic health records and other health technologies	^38^				✓	
Integrating data across IT systems and providers/interoperability		^47^	^44^		✓	
Lack of integrated electronic health records	^31^				✓	
Hospital’s complicated IT system		^35^			✓	
Lack of data standardization		^47^			✓	
Outdated information	^38^				✓	
IT systems do not facilitate the regular measurement of outcomes and cost		^41^			✓	
*Financing*						
Current reimbursement/payment model	^31,38^	^42,45,46^	^44,46^	^46^	✓	
Current healthcare delivery system	^31^				✓	
Lack of appropriate reimbursement for value-added team activities		^47^			✓	
Complexity of financial risk sharing		^40^	^40^	^40^	✓	
Securing fiscal support				^25^	✓	
Significant upfront investment		^40,42,46^	^40^	^40^	✓	
Requires interdepartmental funding		^43^			✓	
*Organizational culture and leadership*						
Requires cultural change		^35,41,42,46^				✓
Most healthcare leaders are not experts in change management		^41^				✓
Existing organizational structure		^41^			✓	
*Workforce*						
Shortage of physicians/providers		^45,46^			✓	
Scheduling complexities		^42^			✓	
Geographic constraints		^43^			✓	
Competency knowledge gaps between healthcare professions	^38^				✓	
Reporting or administrative burden	^38^	^47^			✓	
Primary care providers had concerns about losing patients to the IPU		^36^				✓
Concerns about a potential decline in measured productivity by healthcare professionals in the IPU		^36^				✓
*Communication and coordination*						
Goal alignment			^48^			✓
Access to shared data			^48^		✓	
Sharing of information across practices		^47^			✓	
Competing financial interest of providers as part of the patient’s care cycle			^44^			✓
No alignment of incentives for the providers			^44^			✓
*Commitment*						
Limited institutional resources		^46^			✓	
Lack of legislation promoting and supporting integration				^46^	✓	
Trust between partners			^48^			✓
Resistance to change		^41,43^				✓
Payer-provider collaboration				^25^		✓
*Clinical care*						
Established care patterns		^41^			✓	
Difference across practices, locations		^46^			✓	
*Education*						
Training staff/patients to use the IT platform		^42^			✓	
*Quality improvement*						
Lack of consensus around how to operationalize value measurement		^40^				✓
Acceptance of common measures and benchmarks			^48^			✓
Adoption of quality improvement activities			^48^			✓
Outcome measurements are not used as clinical tools		^41^			✓	
Evidence of cost-effectiveness is lacking		^45^			✓	
Limited evidence on the impact on patient outcomes and costs		^40^			✓	

Abbreviations: IPU, Integrated Practice Unit; IT, information technology. ^25-48^ Represent the article in which the facilitator or barrier was mentioned. ✓ The facilitator and barriers were categorized into functional and normative integration based on how the facilitator or barrier enables the connectivity between the various integration levels.

## Discussion

 The main objective of this study was to provide practical evidence-based recommendations for the implementation of integrated care within a VBHC context, in other words, VBIC. To achieve this aim, we identified how VBIC is defined in current literature, summarized the results of evaluations of the effects of VBIC and summarized the literature regarding the facilitators and barriers for its implementation. Among the articles included in this systematic review, we found one definition for the concept of VBIC. This definition largely overlaps with the principles of VBHC. Two of the core elements of VBHC consist of maximizing patient value by measuring patient achieved outcomes in relation to the amount of money spent to achieve those outcomes, and organizing care around medical conditions and care cycles for a specific patient population. With the exception of patient experience with care, all elements mentioned within the definition of VBIC are also described within the VBHC framework. This confirms that VBIC fits within the larger context of VBHC; the additional focus on patient experiences can be understood from the context of integrated care, which aims to improve patient care and experiences through improved care coordination.^5,9^

 How VBIC fits within the integrated care context is more difficult to distinguish. This is directly related to the complex nature of integrated care; possible similarities between the two concepts are dependent on the used definition of integrated care. However, regardless of the precise definition integrated care is, at its core, an approach to overcome care fragmentation.^8^ Providing accessible, comprehensive and coordinated care is therefore an important element of both integrated care and VBIC.^8,37^

 Furthermore, we aimed to evaluate the effects of VBIC implementation. The results suggest that VBIC interventions may have a positive impact on clinical outcomes, patient-reported outcomes and healthcare utilization. Comparing the effects of VBIC with the effects of integrated care reveals many similarities. Previous systematic reviews on the effects of integrated care suggest that it might have a positive effect on hospital admissions,^18^ readmissions^18^ and patient satisfaction.^18,19^ This was confirmed in our systematic review regarding VBIC.

 At last, this review assessed the facilitators and barriers for the implementation of VBIC. Overall, our findings highlight that healthcare organizations which aim to successfully implement VBIC must invest in a satisfactory IT infrastructure, support and facilitate the implementation of new reimbursement or payment models, remove barriers to cultural change and support strong leadership. A strong leader at the helm of the VBIC intervention can facilitate its implementation by creating an environment in which healthcare providers or organizations are stimulated to trust each other and work together to achieve their common goal. Supportive leadership can thereby also facilitate cultural change. Comparing the factors that influence the successful implementation of VBIC with the facilitating factors for the implementation of VBHC or integrated care reveals many similarities. A well-functioning IT infrastructure, financial support, leadership, and cultural change are also frequently mentioned facilitators and barriers for the implementation of VBHC^49-52^ or integrated care.^16,17,53^

###  Strengths and Limitations

 This is the first systematic review about VBIC. Both empirical and non-empirical studies were included to obtain a broad overview of the current literature on VBIC. No distinction was made as to the type or level of VBIC, which enabled us to provide recommendations for the implementation of VBIC across the whole spectrum of integrated care.

 However, several limitations should also be mentioned. Firstly, this review may not include all relevant articles on VBIC. Although the search strategy aimed to include all articles broadly related to VBIC, we might have missed articles that used different terms to describe VBIC. The absence of registered index terms (eg, MeSH or Emtree) for VBHC, integrated care or VBIC also complicated the search. In addition, due to the exclusion of terms related to IPUs, we might have missed articles that described the implementation of these integrated care components of VBHC. However, since the search strategy included all spelling variation for VBHC and integrated care, we expect the chance of missing a relevant article to be minimal.

 Secondly, since the term VBIC is rarely used, and the definition was still unknown during article selection, the reviewers of this study decided that an article was considered to fit the criteria of VBIC if it described the implementation of integrated care within a VBHC context. This may have led to the inclusion of articles that did not strictly fit the definition of VBIC as provided by Valentijn et al^37^ In addition, the use of the self-determined VBIC criteria may have led to the inclusion of articles that were primarily about VBHC instead of VBIC. Nonetheless, we believe that we screened the articles very carefully and only included articles on VBIC.

 Lastly, our findings on the effectiveness of VBIC interventions should be interpreted with caution. The generalizability is hindered by the limited number of studies that evaluated the effectiveness of a VBIC intervention and the different characteristics of the VBIC intervention. Each study implemented an intervention for a different target population, achieved a different level of integration and used different outcomes measures. Moreover, some articles used a large number of outcome measures and did not define one specific primary outcome measure to evaluate the effect of the intervention. Those articles often found at least one significant reduction or improvement on their outcome measures, which might have been a result of multiple testing.

###  Implications for Research and Practice

 The reviews findings suggest that the concepts VBIC, VBHC and integrated care share a certain level of resemblance. Similarities can be found in the definitions, the effects and the facilitators and barriers for implementation. Resemblance is also inherent to the VBIC interventions; the interventions consist of multiple components related to both VBHC and integrated care. The resemblance between the three concepts, together with the multicomponent interventions, restrict our ability to assess causality between the separate components of the intervention and the results. The added value of VBIC above VBHC or integrated care, therefore, remains unclear. This raises the question if VBIC is substantially different from VBHC and integrated care, and if a separate definition for VBIC provides additional value. Evidently, it would be prudent to further investigate the resemblance and possible distinction between VBIC, VBHC and integrated care.

 Moreover, clear guidelines should be developed to facilitate the implementation and evaluation of VBIC, VBHC or integrated care interventions. Those guidelines should include recommendations on research design and the selection of outcome measures. Based on our findings we recommend that further research should evaluate the effectiveness of an implementation using a randomized clinical trial, stepped-wedge or cohort design (ie, a study design with a control group), to ensure that researchers are able to assess causality. In addition, we recommend that researchers evaluate the effects of one intervention at a time and critically assess the outcome measures needed to measure the effect, and define those upfront. All outcome measures must be relevant to the intervention and should be included if a change is expected to occur after implementation, to limit the burden of outcome collection and the possibility of multiple testing.

 Furthermore, our findings provide an overview of all possible factors that influence the successful implementation of VBIC. Healthcare organizations wishing to implement VBIC can use this overview to create the ideal environment for implementation and increase the chance of a successful implementation. Further research could be performed to identify the underlying mechanism of the influencing factors. Such research will increase the understanding of why a certain factor facilitates or hinders VBIC and provide more insight into the intricacies of VBIC implementation.

 An increased understanding of the facilitating or hindering factors for VBIC is also necessary to enable organization to achieve sustainable healthcare services. To maximize patient value across the whole cycle of care, care needs to be integrated on organizational or preferably system level.^13,54,55^ Many VBIC initiatives, however, focus on achieving clinical or professional integration. These initiatives are often driven by a bottom-up approach, advocated by healthcare professionals with the aim to deliver more patient-centred care. The transformation to system level integration requires both a bottom-up and top-down approach; it requires collaboration between professionals, organizations, governments, and healthcare insurers. This collaboration needs to be supported by (national) policies and regulations, and by functional and normative integration mechanisms such as a shared mission and adequate financial, management and information systems. Only by facilitating collaborations and removing the barriers for integration will healthcare organizations be able to achieve true VBIC on a system level.

## Conclusions

 This systematic review found that the concept of VBIC is not well defined in current literature. Only one definition of VBIC was found which described VBIC as patients’ achieved outcomes and experience of care in combination with the amount of money spent by providing accessible, comprehensive and coordinated services to a target population. The effect of VBIC seems promising and comparable to integrated care or VBHC, but the exact interpretation of effect evaluations is challenged by the precedence of multicomponent interventions, multiple testing and generalizability issues. For successful implementation of integrated care within a VBHC context, it is imperative that healthcare organizations consider investing in appropriate IT infrastructure and the development and implementation of new reimbursement models.

## Acknowledgements

 We would like to thank our consortium members: I.L. Abma, C.T.B. Ahaus, R.J. Baatenburg de Jong, M.C. de Bruijne, M.C. Dorr, E.A.C. Dronkers, H.J. van Elten, L. Haverman, J.G.M. Jelsma, M. Leusder, M.M. van Muilekom, M.P.J. Offerman, T.S. Reindersma, K.S. van Hof, and P.J van der Wees for their contribution.

 We also wish to thank W.M. Bramer from the Erasmus MC Medical Library for developing and updating the search strategies.

## Ethical issues

 Not applicable.

## Competing interests

 Authors declare that they have no competing interests.

## Funding

 This work was supported by Dutch Ministry of Health, Welfare and Sport [grant number: 330843].
